# Moderate-intensity exercise allows enhanced protection against oxidative stress-induced cardiac dysfunction in spontaneously hypertensive rats

**DOI:** 10.1590/1414-431X20198009

**Published:** 2019-05-16

**Authors:** Chunjuan Mi, Xinghua Qin, Zuoxu Hou, Feng Gao

**Affiliations:** 1 School of Life Science and Technology, Shaanxi Normal University, Xi'an, China; 2 Department of Physical Education, Xi'an University of Science and Technology, Xi'an, China; 3 School of Aerospace Medicine, Fourth Military Medical University, Xi'an, China

**Keywords:** Exercise training, SHR, Oxidative stress, SIRT3/SOD2, Mitochondria function

## Abstract

The progression of myocardial injury secondary to hypertension is a complex process related to a series of physiological and molecular factors including oxidative stress. This study aimed to investigate whether moderate-intensity exercise (MIE) could improve cardiac function and oxidative stress in spontaneously hypertensive rats (SHRs). Eight-week-old male SHRs and age-matched male Wistar-Kyoto rats were randomly assigned to exercise training (treadmill running at a speed of 20 m/min for 1 h continuously) or kept sedentary for 16 weeks. Cardiac function was monitored by polygraph; cardiac mitochondrial structure was observed by scanning electron microscope; tissue free radical production was measured using dihydroethidium staining. Expression levels of SIRT3 and SOD2 protein were measured by western blot, and cardiac antioxidants were assessed by assay kits. MIE improved the cardiac function of SHRs by decreasing left ventricular systolic pressure (LVSP), and first derivation of LVP (+LVdP/dt_max_ and −LVdP/dt_max_). In addition, exercise-induced beneficial effects in SHRs were mediated by decreasing damage to myocardial mitochondrial morphology, decreasing production of reactive oxygen species, increasing glutathione level, decreasing oxidized glutathione level, increasing expression of SIRT3/SOD2, and increasing activity of superoxide dismutase. Exercise training in SHRs improved cardiac function by inhibiting hypertension-induced myocardial mitochondrial damage and attenuating oxidative stresses, offering new insights into prevention and treatment of hypertension.

## Introduction

The World Health Organization estimates that hypertension affects approximately 25% of adults worldwide (1). Exercise is a recommended adjunct to many pharmaceutical antihypertensive therapies. There is clear evidence that physical activity may improve cardiovascular function and prevent cardiovascular disease (2), partly through regulating oxidative balance and decreasing inflammation. However, the effects of chronic exercise on the development of hypertension-induced cardiac redox status remain unknown.

Although the pathogenesis of hypertension can be influenced by multiple factors (3), the development of cardiac mitochondrial dysfunction contributes to the development of hypertension. Importantly, oxidative stress has been shown to play a crucial role in the development of mitochondrial dysfunction and alter a variety of cellular signaling cascades and cellular functions (4). Exercise has been reported to produce adaptive responses to oxidative stress, which has been studied primarily in skeletal muscles, but also in the heart.

SIRT3 has become a novel target pursued substantially for its involvement in regulation of aging and age-related diseases, possibly due to its well-known association with exceptionally long lifespan in humans as well as its mitochondrial localization (5). Superoxide dismutase (SOD) is the primary mitochondrial antioxidant enzyme. SIRT3 directly deacetylates SOD2 in mitochondria, significantly enhancing its ability to scavenge reactive oxygen species (ROS) (6,7). Exercise in mice increased SIRT3 protein expression in cardiac and triceps muscles (8). However, less is known about the effects of chronic exercise on the relationship between cardiac mitochondrial function and SIRT3/SOD2 signaling in the hypertensive condition.

We hypothesized that regular chronic moderate-intensity exercise (MIE) improves cardiac oxidative state in the progression of hypertension of spontaneously hypertensive rats (SHRs). Furthermore, the beneficial effects of MIE in SHRs would be mediated by upregulated myocardial antioxidant molecules, including SIRT3/SOD2. Results from these studies will help us further understand the mechanism by which exercise ameliorates hypertension.

## Material and Methods

The experiments were performed in accordance with the National Institutes of Health Guidelines for the Use of Laboratory Animals and were approved by the Fourth Military Medical University Animal Care Committee (China).

### Experimental animals and treatment

Eight-week-old male SHRs were divided into 2 groups: sedentary group (SHR+Sed, n=5) and exercising group (SHR+Exe, n=5). Age-matched male Wistar-Kyoto (WKY) rats were used as the controls (WKY+Sed, n=5; WKY+ Exe, n=5). All rats were obtained from Vital River (China). All groups were provided the standard diet (Fourth Military Medical University Animal Care Committee, China) and water. The room was maintained at 22±2°C in a controlled 12-h light/dark cycle (light period from 08:00 a.m. to 20:00 p.m.) with 45–55% relative humidity. Rats in the SHR+Exe and WKY+Exe groups ran on a motor-driven treadmill (model JD-PT, Jide Education Instruments Co, China) at a velocity of 20 m/min for 1 h, corresponding to moderate exercise. The treadmill speed was modified according to the protocols of Husain (9). In contrast, the WKY+Sed and SHR+Sed groups did not receive any exercise program. Forty-eight hours after the last exercise session of the 16-week-long protocol, all rats were euthanized immediately following the anesthetization using 3% pentobarbital sodium (Merck; Germany) for approximately 10 min (until the mouse was immobile). The cardiac tissues were immediately isolated for the experiments described below.

### Cardiac function assessment

For the assessment of cardiac function, a catheter was inserted into the left ventricle through the right carotid artery to measure the left ventricular pressure (LVP). Echocardiogram and LVP were simultaneously recorded on a polygraph (RM-6200C; China). Left ventricular systolic pressure (LVSP), left ventricular end diastolic pressure (LVEDP), and the instantaneous first derivation of LVP (+LVdP/dt_max_ and −LVdP/dt_max_) were derived by computational algorithms.

### Ultrastructure detection of cardiac mitochondria

Cardiac tissues were washed with cold phosphate buffered saline (PBS), and fixed with EM Grade 4% glutaraldehyde in 0.1 M cacodylate buffer (pH 7.4). Fixed tissues were incubated with 1% osmium tetroxide in cacodylate buffer for 2 h and processed for embedding. Ultra-thin sections were placed on 200 mesh copper grids, and stained with uranyl acetate and lead citrate. Samples were visualized using a JEM-1220 Jeol transmission electron microscopy (JEM, USA), and micrographs were taken using a Gatan digital micrograph (Gatan Microscopy, USA). All image-based measurements were performed using the corresponding workstation at the Fourth Military Medical University. Random images (n=15) from each sample (n=4) were taken for further analysis.

### Reactive oxygen species (ROS) detection in the cardiac tissue

The oxidation-sensitive fluorescent dye dihydroethidium (DHE, Vigorous, R001, China) was used for *in situ* evaluation of ROS content. Rats were sacrificed 48 h after the last exercise and cardiac tissues were harvested and embedded in optimum cutting temperature (OCT) medium. Sections (14 μm) were placed on glass slides and allowed to reach equilibrium (30 min at 37°C in PBS). The sections were then incubated with DHE (10 μM DHE in PBS) at 37°C for 30 min in the dark; control sections received an identical volume of PBS. Fluorescent images were captured by Zeiss LSM510 Meta laser scanning microscopy (Nikon, Japan). The DHE fluorescence intensity was observed in 10 images of every 3 to 4 hearts in each group. All images were processed equally and subjected to background corrections.

### Measurement of GSH and GSSG levels

Content levels of glutathione (GSH) and oxidized glutathione (GSSG) were determined with specific assay kits (S0053, Beyotime, China). In brief, freshly isolated cardiac tissues (0.5 mg) were rapidly centrifuged at 10,000 *g* for 5 min at 4^o^C. For GSH determination, 100 mL of supernatant was added to 1.8 mL phosphate buffer and 100 mL o-phthalaldehyde (OPT). After thoroughly mixing and incubation at room temperature for 15 min, the solution was transferred to a quartz cuvette and its fluorescence intensity was assessed at 412 nm emission and excitation wavelengths. For GSSG determination, 0.4 mL of supernatant was taken, then 0.6 mL (0.5 M, pH 0.5) of dilute phosphoric acid buffer was added, followed by adding 40 μL of 2-vinyl pyridine and 1-h incubation at 25°C. The solution was transferred to a quartz cuvette and the fluorescence was measured at 412 nm emission and excitation wavelengths. GSH and GSSG contents were determined from comparisons with linear standard curves.

### Western blot analysis

To determine the expression of SIRT3 and SOD2, the standard SDS–PAGE western blot technique was performed as described previously (10). Proteins were prepared according to the kit protocol. Protein concentration was determined using a BCA assay kit (Beyotime). Proteins were separated on 10% SDS-polyacrylamide gels for SIRT3 (CST, USA), SOD2 (Epitomics, USA), and GAPDH (Abcam, USA). Antibodies were incubated with proteins overnight at 4°C, and subsequently incubated with the corresponding secondary antibodies at room temperature for 1 h. The blots were visualized with ChemiDocXRS (Bio-Rad Laboratory, USA).

### SOD2 enzyme activity assay

Cell samples were briefly sonicated after centrifugation at 12,000 *g* for 5 min at 4°C. Immediately after preparation and concentration assessment, SOD2 activity was detected with an assay kit (Cell Technology, USA), using a water-soluble tetrazolium salt that produced a water-soluble formazan dye upon reduction with a superoxide anion. Accordingly, reduction rate was regarded as the measurement of SOD activity present in the experimental samples.

### Statistical analysis

The Shapiro-Wilk test was used to assess data normality, and W values for all experimental data were above 0.9 with P>0.05, indicating that the null hypothesis was accepted and the data were normally distributed. All data are reported as means±SE. One-way analysis of variance (ANOVA) followed by Newman-Keuls multiple comparison test or unpaired *t*-test (GraphPad Prism software, USA) was used to determine statistical differences between the mean values of the groups. The results were considered significant at P<0.05.

## Results

### MIE improved cardiac function in SHRs

**Figure 1.**
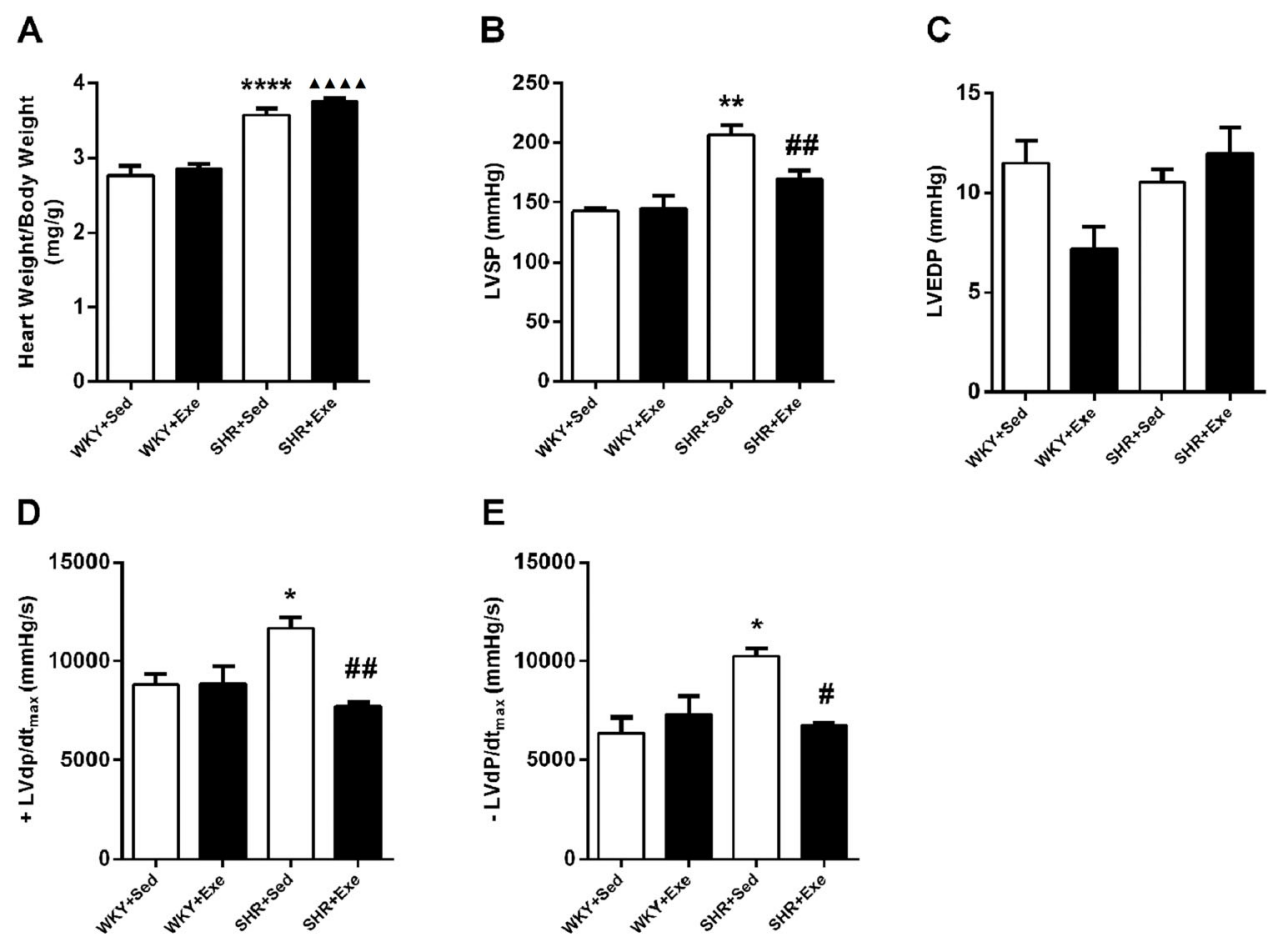
Moderate-intensity exercise improved cardiac function in spontaneous hypertensive rats (SHRs). Eight-week-old male SHRs and age-matched male Wistar-Kyoto (WKY) rats were randomly assigned to exercise training (Exe) or kept sedentary (Sed) for 16 weeks. The graph shows changes of heart weight/body weight ratio (**A**) and indexes of cardiac function including LVSP (**B**), LVEDP (**C**), +LVdP/dt_max_ (**D**), and −LVdP/dt_max_ (**E**) measured in WKY+Sed, WKY+Exe, SHR+Sed and SHR+Exe groups. Data are reported as means±SE (n=4 for **A** and n=3 for **B**-**E**). *P<0.05, **P<0.01, ****P<0.0001 *vs* WKY+Sed group; ^▲▲▲▲^P<0.0001 *vs* WKY+Exe group; ^#^P<0.05, ^##^P<0.01, *vs* SHR+Sed group (ANOVA). LVSP: left ventricular systolic pressure; LVEDP: left ventricular end diastolic pressure; ±LVdP/dt_max_: positive or negative instantaneous first derivation of left ventricle pressure.

As shown in Figure 1, 16-week MIE did not lead to changes of heart weight/body weight (HW/BW) ratio and indexes of cardiac function including LVSP, LVEDP, +LVdP/dt_max_, and −LVdP/dt_max_ in WKY rats, indicating MIE does not affect cardiac function in non-hypertensive rats. However, compared with the WKY+Sed group, there were increases of HW/BW ratio (P<0.0001), LVSP (P<0.01), +LVdP/dt_max_ (P<0.05), and −LVdP/dt_max_ (P<0.05) in SHR+Sed group, suggesting blood pressure elevation impaired cardiac functions. Importantly, MIE significantly decreased +LVdP/dt_max_ (P<0.01), −LVdP/dt_max_ (P<0.05), and LVSP (P<0.01) in SHRs; while HW/BW ratio and LVEDP showed no significant differences in SHR+Sed group compared with SHR+ Exe group. These results indicated that chronic MIE improved cardiac systolic and diastolic function in SHRs.

### MIE decreased damage to myocardial mitochondrial morphology in SHRs

**Figure 2.**
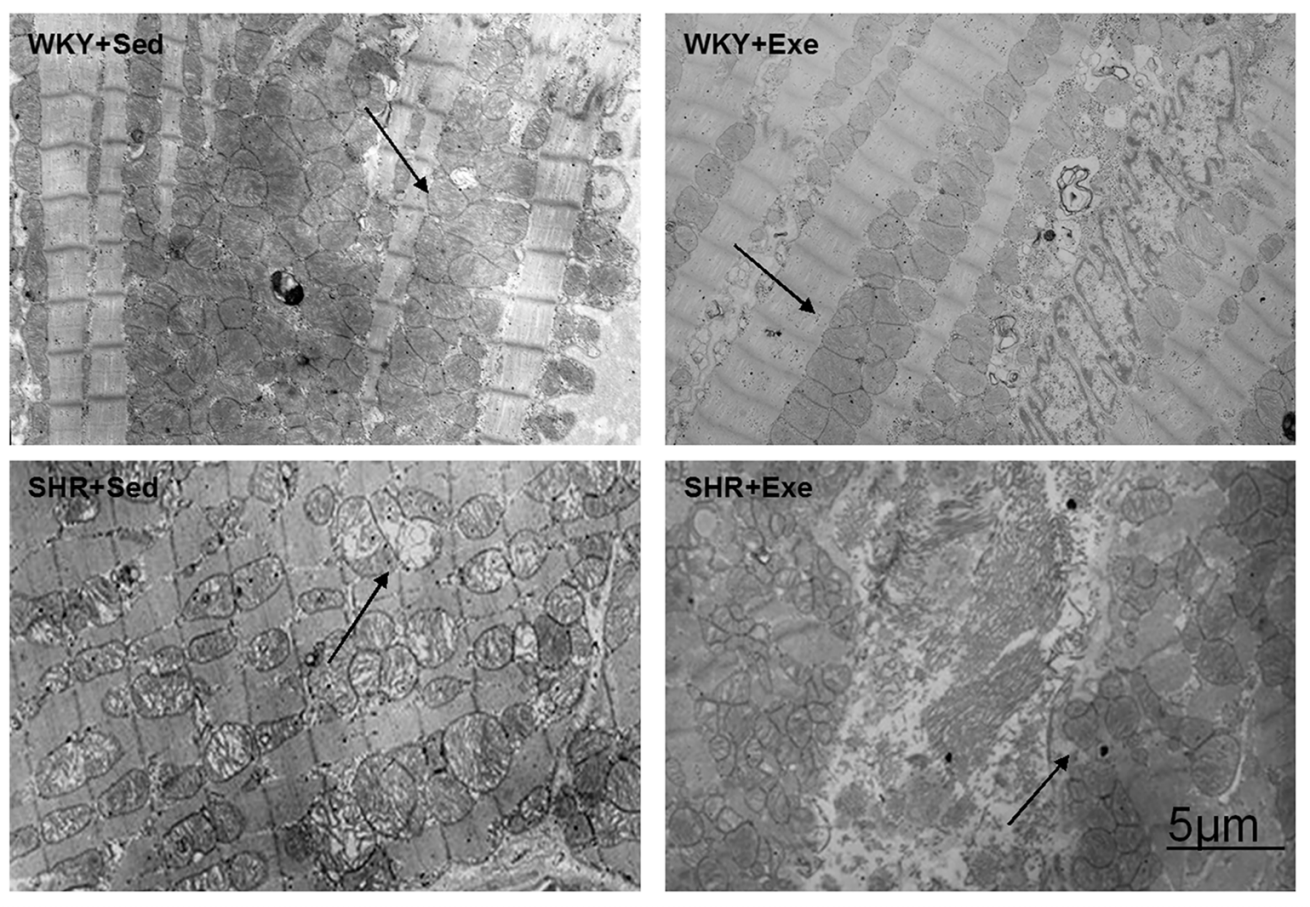
Moderate-intensity exercise (MIE) ameliorated damage to myocardial mitochondrial morphology in spontaneously hypertensive rats (SHRs). Cardiac mitochondrial structure was observed in Wistar-Kyoto rats sedentary or exercised (WKY+Sed, WKY+Exe), and SHR+Sed and SHR+Exe groups after 16 weeks of MIE by scanning electron microscopy. Arrows: myocardial mitochondria. Scale bar: 5 µm.

By electron microscopy, SHRs showed significant damage to the mitochondria inner membrane and cisterna (Figure 2). The damaged area identified by the ratio of vacuous area within a mitochondrion to the whole mitochondrion was significantly higher in the SHRs than in the WKY rats. The ratio was ameliorated by exercise.

### MIE reduced cardiac ROS production in WKY rats and SHRs

**Figure 3.**
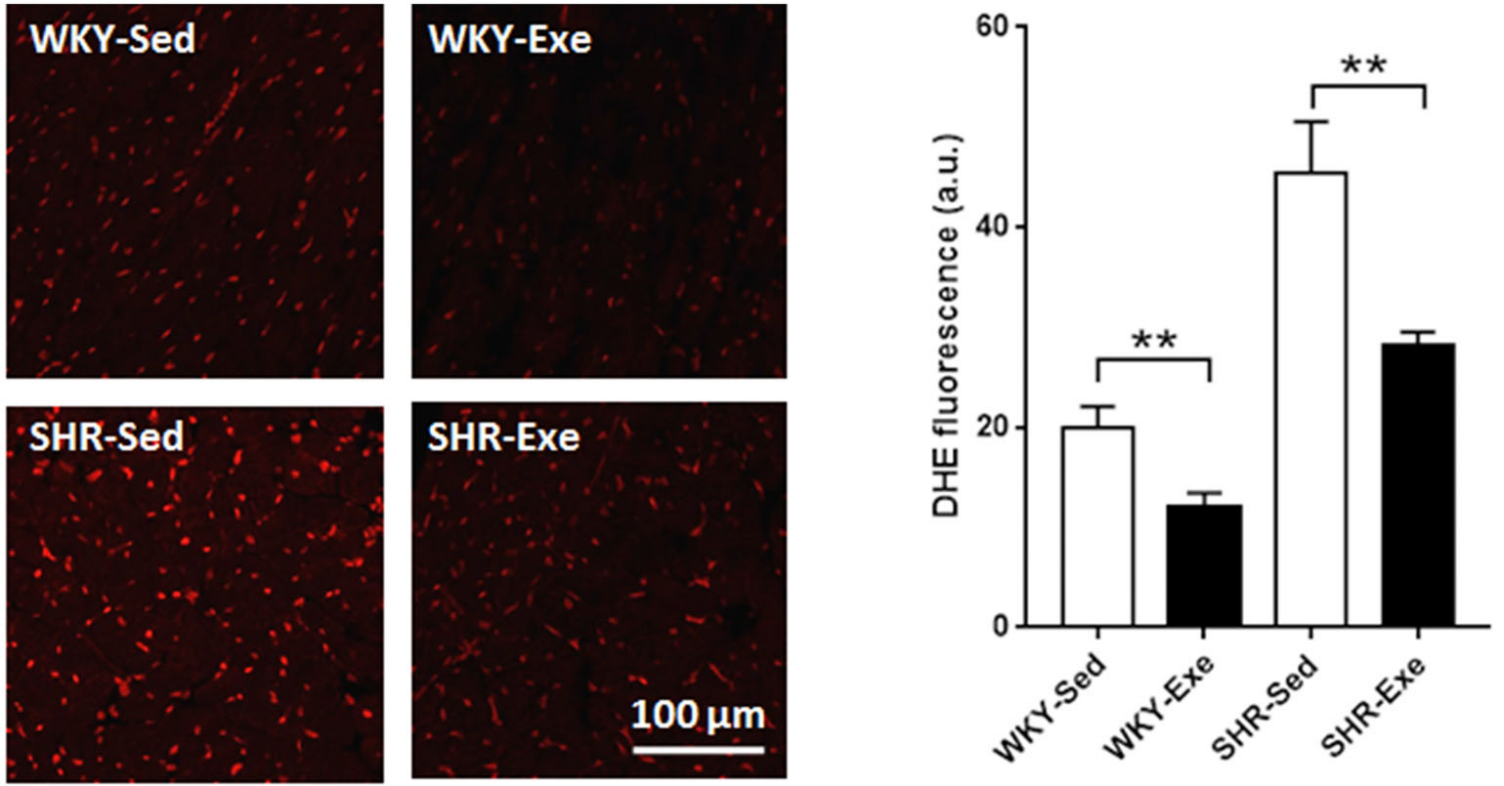
Cardiac reactive oxygen species levels were determined in sedentary or exercised Wistar-Kyoto rats (WKY+Sed, WKY+Exe) and in sedentary or exercised spontaneously hypertensive rats (SHR+Sed and SHR+Exe) by dihydroethidium staining after 16 weeks of moderate-intensity exercise. Representative images and statistical histogram are shown. Data are reported as means±SE (n=4). **P<0.01 (ANOVA). Scale bar: 100 µm.

As shown in Figure 3, DHE staining was significantly lower in the WKY+Exe group compared to the WKY+Sed group (P<0.05). Compared with the WKY+Sed group, there was a significant increase of ROS level in the SHR+Sed group (P<0.0001). However, cardiac ROS levels were significantly decreased in the SHR+Exe group compared to the SHR+Sed group (P<0.0001).

### MIE increased cardiac GSH, but decreased GSSG levels in WKY rats and SHRs

**Figure 4.**
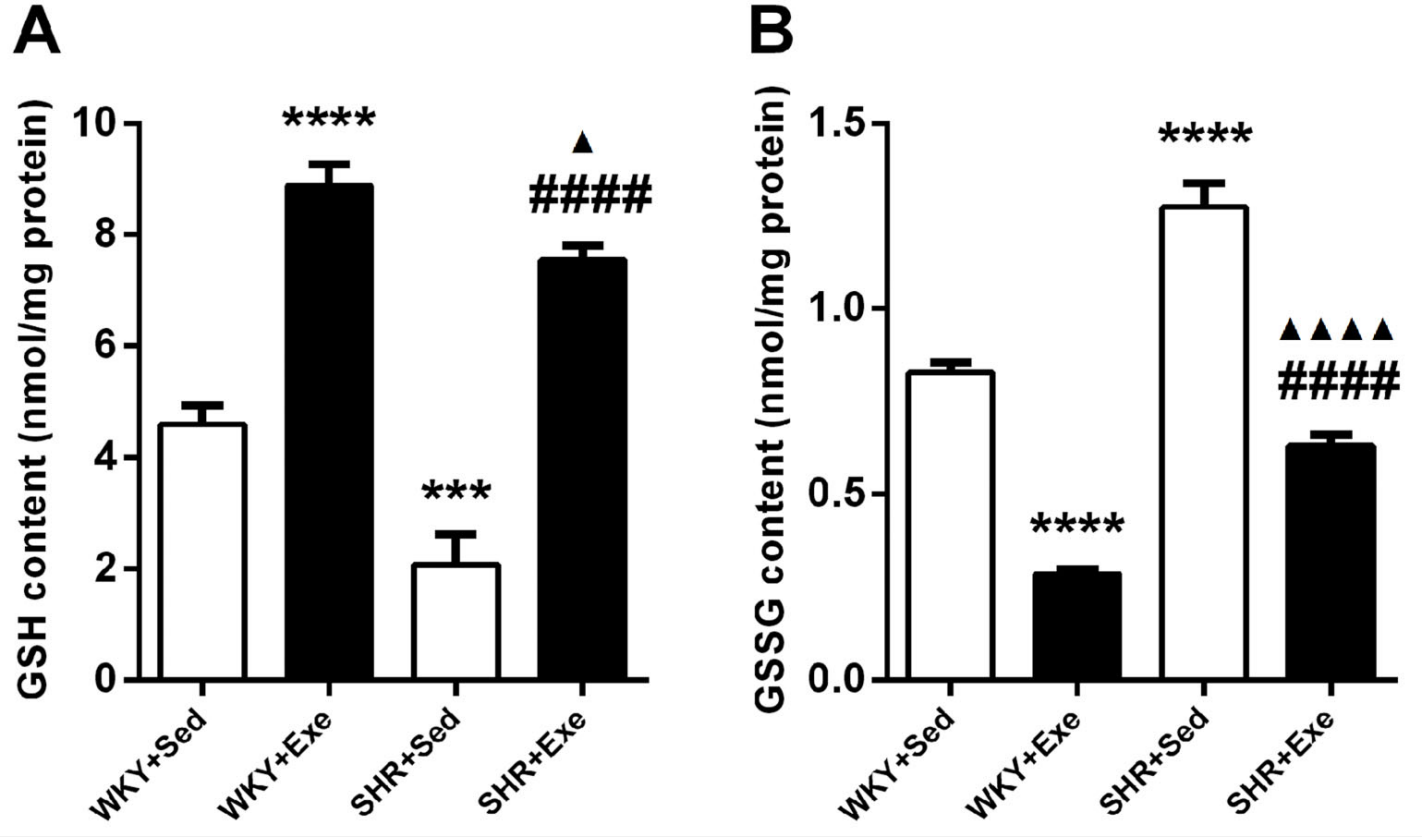
Cardiac GSH (**A**) and GSSG (**B**) levels were measured in sedentary or exercised Wistar-Kyoto rats (WKY+Sed, WKY+Exe) and in sedentary or exercised spontaneously hypertensive rats (SHR+Sed and SHR+Exe) after 16 weeks of moderate-intensity exercise. Results are reported as means±SE (n=4 for **A** and n=5 for **B**). ***P<0.001, ****P<0.0001 *vs* WKY+Sed group; ^▲^P<0.05, ^▲▲▲▲^P<0.0001 *vs* WKY+Exe group; ^####^P<0.0001 *vs* SHR+Sed group (ANOVA). GSH: reduced glutathione; GSSG: oxidized glutathione.

As shown in Figure 4, 16-week MIE increased GSH level (P<0.0001), but decreased GSSG level (P<0.0001) in WKY rats. Compared with the WKY+Sed group, there was a decrease of GSH level (P<0.001) and an increase of GSSG level (P<0.0001) in the SHR+Sed group. However, GSH content significantly increased (P<0.0001), but GSSG content decreased (P<0.0001) in the SHR+ Exe group compared to the SHR+Sed group.

### MIE increased cardiac expression of SIRT3 and SOD2, and SOD2 activity

**Figure 5.**
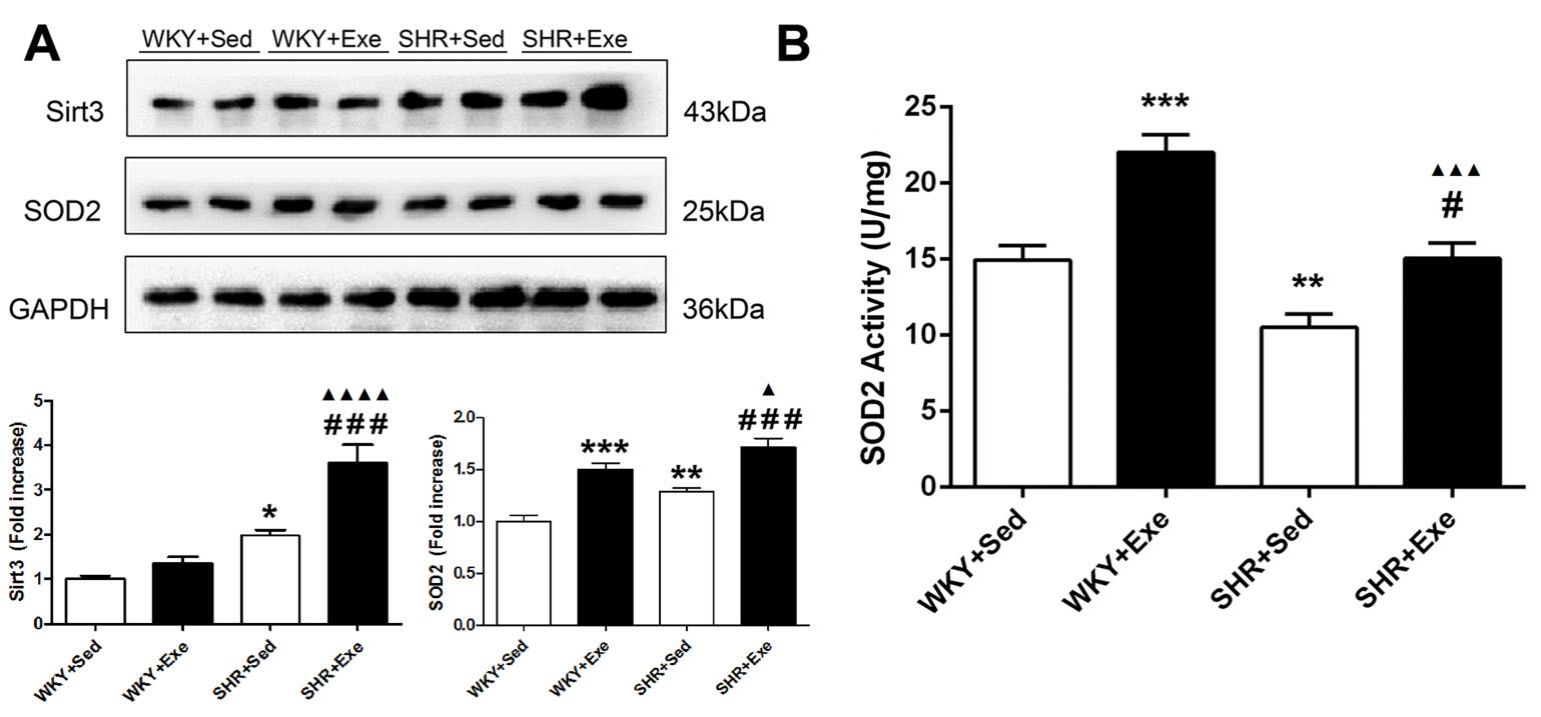
A, Representative blots of SIRT3 in cardiac tissues in sedentary or exercised Wistar-Kyoto rats (WKY+Sed, WKY+Exe) and in sedentary or exercised spontaneously hypertensive rats (SHR+Sed, SHR+Exe) after 16 weeks of moderate-intensity exercise are presented in the upper panel. Quantification of immunoreactive bands is presented in the lower panels. **B**, Superoxide dismutase (SOD2) activity in cardiac tissues of the same groups. Data are reported as means±SE (n=4 for **A** and n=5 for **B**). *P<0.05, **P<0.01, ***P<0.001 *vs* WKY+Sed group; ^▲^P<0.05, ^▲▲▲^P<0.001, ^▲▲▲▲^P<0.0001 *vs* WKY+Exe group; ^#^P<0.05, ^###^P<0.001 *vs* SHR+Sed group (ANOVA).

Western blot analysis showed that SIRT3 protein levels were significantly increased in the SHR+Exe group compared with the WKY+Exe (P<0.0001) and SHR+ Sed groups (P<0.001). SOD2 protein levels were also elevated in the SHR+Exe group compared with the WKY+Exe (P<0.05) and SHR+Sed groups (P<0.001) (Figure 5A). As shown in Figure 5B, SOD2 activity was also increased in the SHR+Exe group compared with the SHR+Sed group (P<0.05).

## Discussion

Hypertension is a major risk factor for myocardial injury characterized by cardiac hypertrophy and fibrosis. In this study, we investigated the effects of chronic MIE and possible mechanisms of the improved effects on cardiac oxidative stress in SHRs. Our results showed that regular MIE improves the cardiac function of SHRs by decreasing LVSP, +LVdP/dt_max_, and −LVdP/dt_max_. Exercise-induced beneficial effects in SHRs were mediated by reducing ultrastructural damages to myocardial mitochondria, increasing GSH level, decreasing GSSG level, and increasing expression of SIRT3 and SOD2, as well as enhancing the activity of SOD2. These findings provided evidence for the involvement of redox homeostasis and SIRT3/SOD2 signaling in exercise-induced cardiac improvements in SHRs.

Our findings on improved cardiac function from exercise in SHRs are consistent with our previous report (10). We observed that mitochondrial morphological features of rats from the SHR+Exe group changed in contrast to that of the SHR+Sed group.

Oxidative stress produced by overproduction of ROS has been considered to be involved in the development and progression of hypertension and hypertension-induced cardiac injury (11,12). Consistent with previous studies, we found that the level of ROS was higher in the SHR+Sed group than in the WKY+Sed group. Interestingly, MIE significantly reduced cardiac ROS production in both WKY rats and SHR. Moreover, exercise training significantly increased GSH content and decreased GSSG content of cardiac tissue. The above results indicate that redox status was altered and oxidative stress was decreased after MIE. Indeed, exercise training has been reported to produce adaptive responses to oxidative stress, as studied primarily on skeletal muscles and cardiac tissue after 8 weeks of aerobic training (13).

In our investigation, we found that 16-week treadmill training exercise increased SIRT3 and SOD2 protein expression, which was associated with improved cardiac redox state. An important question raised in this study was whether our results were solely due to the improved cardiac function caused by exercise or due to the improved mitochondrial function. Previous data have demonstrated that cardiac functional and structural changes can occur in response to alterations in mitochondrial function. The heart produces and utilizes more energy than any other organ, and more than 90% of its energy is produced from mitochondrial respiration (14).

*SIRT3* gene is highly expressed in brain, heart, liver, kidney, testis, and muscles (15,16), and the SIRT3 protein is localized in the mitochondrial matrix. SIRT3 has been found to block development of cardiac hypertrophy and protect cardiomyocytes from oxidative stress-mediated cell death. In a clinical study, it was reported that individuals with a sedentary lifestyle at the age of 60 or above had 40% reduced SIRT3 levels compared with younger subjects. After endurance exercise, the health benefits of older patients were accompanied by elevated levels of SIRT3 (17). In our present study, SHR+Sed subjects had higher SIRT3 expression than WKY+Sed subjects despite no statistical significance, which is consistent with previous reports. Interestingly, we observed a significant elevation in SIRT3 expression and a decrease in ROS level in SHR+ Exe compared to SHR+Sed. Emerging evidence indicates that exercise can decrease oxidative stress and we believe that exercise-induced activation of SIRT3 is a key step of this defense in the condition of hypertension.

SOD2 plays an essential role in mitochondrial function and protection by limiting oxidative stress directly via its antioxidant effects. Thus, SOD2, the major mitochondrial antioxidant scavenger, was investigated in the present study. Our results demonstrate that SOD2 levels were increased in the heart following the 16-week treadmill training. These results were consistent with a previous report (18). Han C and Someya (19) found that the ability of SOD2 to reduce cellular ROS and promote oxidative stress resistance was greatly enhanced by SIRT3 on the condition of calorie restriction. In the present study, we detected that the activation of SOD2 may be linked to the upstream activation of SIRT3 in the heart when SHRs subjected to long-term moderate intensity treadmill training, which is crucial for the maintenance of redox control during exercise.

In summary, the present study showed that 16-week MIE induced positive adaptations in cardiac function accompanied with improved redox status of SHRs, characterized by decreasing +LVdP/dt_max_ and −LVdP/dt_max_, and ROS levels, and increasing GSH and GSSG levels, and SIRT3 and SOD2 protein expression. For the first time, we showed that exercise can improve cardiac redox status of SHRs via upregulation of SIRT3/SOD2 expression. In other words, increased antioxidant defense induced by long-term training adapted well to mitochondrial dysfunction and thus it preserved cardiac mitochondria redox status. Our data also provide a reference for further studies on mechanisms by which regular exercise may alter cardiac mitochondrial metabolism and protect against hypertension-induced oxidative stress.

The exclusion of all the possibilities pertaining to the improvement of cardiac function in hypertensive rats is not possible. For instance, a reduction in cardiac afterload could result in attenuation of these parameters. Moreover, it is possible that a smaller concentric cavity in the exercised rats could generate a smaller dP/dt as a function of Laplace's law, suggesting that geometry of the LV, in particular the diameter and wall thickness of the LV, should be comprehensively explored in future investigations.
